# Characterization of a High PM_2.5_ Exposure Group in Seoul Using the Korea Simulation Exposure Model for PM_2.5_ (KoSEM-PM) Based on Time–Activity Patterns and Microenvironmental Measurements

**DOI:** 10.3390/ijerph15122808

**Published:** 2018-12-10

**Authors:** Yunhyung Hwang, Jaehoon An, Kiyoung Lee

**Affiliations:** 1 Department of Environmental Health Sciences, Graduate School of Public Health, Seoul National University, Seoul 08826, Korea; hyh@snu.ac.kr; 2 Department of Public Health Sciences, Graduate School of Public Health, Seoul National University, Seoul 08826, Korea; lyra57@snu.ac.kr; 3 Institute of Health and Environment, Seoul National University, Seoul 08826, Korea

**Keywords:** personal exposure, fine particle, national representative level, time-activity pattern, microenvironment, exposure assessment, high exposure group, Korean model

## Abstract

The Korea Simulation Exposure Model for fine particulate matter (PM_2.5_) (KoSEM-PM) was developed to estimate population PM_2.5_ exposure in Korea. The data were acquired based on 59,945 min of the actual microenvironmental PM_2.5_ measurements and on the time–activity patterns of 8072 residents of Seoul. The aims of the study were to estimate daily PM_2.5_ exposure of Seoul population, and to determine the characteristics of a high exposure group. KoSEM-PM estimated population exposures by applying the PM_2.5_ distribution to the matching time–activity patterns at 10-min intervals. The mean personal PM_2.5_ exposure level of the surveyed subjects in Seoul was 26.0 ± 2.7 µg/m^3^ (range: 21.0–40.2 µg/m^3^) in summer. Factors significantly associated with high exposure included day of the week, age, industry sector, job type, and working hours. Individuals surveyed on Saturdays were more likely to be in the high exposure group than those surveyed on weekdays and Sundays. Younger, non-office-working individuals with longer working hours were more likely to be in the high exposure group. KoSEM-PM could be a useful tool to estimate population exposure levels to other region in Korea; to expand its use, microenvironmental measurements are required for other region in Korea.

## 1. Introduction

Exposure to particulate matter (PM) is associated with respiratory health, cardiovascular health, adverse birth outcomes, hospitalization, and mortality [1,2,3,4,5]. The International Agency for Research on Cancer (IARC) classified PM from outdoor air pollution as a human carcinogen (IARC Group 1), based on observational evidence of carcinogenicity in humans and experimental animals, in addition to strong mechanistic evidence [6].

Personal exposure to fine particulate matter (PM_2.5_) can be determined by direct measurements. Although such measurements can yield accurate data, there are time and resource limitations. Most personal exposure monitoring studies have focused on specific groups of subjects, such as medical patients [7,8], children [9,10,11,12], seniors [13,14], and workers [11,15,16]. Thus, these studies are not generalizable to the general population. Personal exposure monitoring data are usually based on a small number of subjects and cross-sectional measurements. Personal exposure levels for PM_2.5_ and PM_1.0_ were measured simultaneously in 30 subjects in Sweden [17]. Elsewhere, personal PM_2.5_ exposure levels were measured in 17 volunteers over two periods in Edinburgh, Scotland [18]. Personal PM_2.5_ exposures of 9–13 subjects were measured in eight districts of Guangzhou, China during the winter [19]. The relationships between indoor and outdoor PM_2.5_ concentrations and personal PM_2.5_ exposures were determined in Windsor, Ontario, Canada [20].

Another method to determine personal exposure uses an indirect approach based on microenvironmental concentrations and the time spent in different microenvironments. The Stochastic Human Exposure Dose Simulation–Particulate Matter (SHEDS-PM) calculated the residential PM concentration via a mass balance model using data on ambient outdoor PM concentrations and physical factors (e.g., air exchange, penetration, and deposition), as well as the emissions from indoor PM sources (e.g., smoking and cooking) [21]. The PM concentrations in eight nonresidential microenvironments were calculated by regression analysis of available indoor and outdoor measurement data. SHEDS-PM was applied to the population of Philadelphia using spatially and temporally interpolated ambient PM_2.5_ data from the 1990 and 1992–1993 US Census, for each census tract in Philadelphia [21]. The tracts showed substantial variability in daily total PM_2.5_ exposures (median = 20 µg/m^3^; 90th percentile = 59 µg/m^3^) [21].

Hazardous Air Pollutant Exposure Model (HAPEM) was used to examine the relationship between modeled personal exposure levels and outdoor concentrations of a large number of particulate pollutants [22]. The outdoor concentrations were used to calculate the concentration by microenvironment, with adjustment for penetration, proximity, and additive factors, while also accounting for variation in emission sources among microenvironments. HAPEM used data from an air quality dispersion model and an inhalation exposure model; the predicted chronic exposure concentrations for outdoor air pollution were lower than the modeled values by approximately 60% for most particulate pollutants. Personal exposure tended to be higher near major emission sources, and when individuals were exposed to pollutants during daily activities, as revealed by their time-activity patterns.

The exposure model for individuals (EMI) was developed using the PM panel study data consisted of 37 participants in North Carolina, USA [23]. The model predicted residential air exchange rate (AER), infiltration factors, indoor concentrations, personal exposure factors, and personal exposures from the outdoor concentrations, questionnaires, weather, and time-location information. The predictions were compared to 591 daily measurements from 31 participants. Median absolute difference was 20% (1.8 μg/m^3^) for personal exposures.

Three exposure models (microenvironmental model, central-site model, and time-space model) for the personal exposure of children to PM_2.5_ were developed using the panel study consisted of 20 asthmatic children in California, USA [24]. The estimated exposures were 27.1 ± 31.5 μg/m^3^. A time–space model based on the PM from fixed site monitoring station and factors representing time activity patterns, season and distance from home showed the highest R^2^ of 0.41.

The Korea Simulation Exposure Model for PM_2.5_ (KoSEM-PM) estimates PM exposure by taking account of microenvironmental concentrations and time–activity patterns. Unlike other models, this study used the time-activity patterns of population from the national survey with stratified subject selection, and the directly measured PM_2.5_ concentration in microenvironment was applied for estimating daily PM_2.5_ exposures. The purpose of this study was to determine the factors associated with the high PM_2.5_ exposures of the population in Seoul.

## 2. Materials and Methods

### 2.1. Microenvironmental Concentration Measurements

The PM_2.5_ data used in this study were measured in Seoul. Seoul is the largest metropolis and capital of South Korea. The city is located in the central western part of the Korean peninsula. The area of Seoul is 605 km^2^. As of 2017, the population was 9.78 million. The microenvironmental PM_2.5_ concentration was measured at 1-min intervals by a portable aerosol spectrometer (Model 1.109; Grimm, Ainring, Germany), worn with the inlet of the monitor positioned as close to the breathing zone as possible. The performance evaluations of the instrument were conducted in previous studies [25,26]. For all measurements, the datalogging interval of the spectrometer was set to 1 min. The sampling flow rate was 1.2 L/min. A gravimetric correction factor was applied using the particle weight obtained from 47-mm polytetrafluoroethylene filters during the monitoring runs. The mean correction factor was 1.1 ± 0.1. A zero calibration of the spectrometer was conducted before each measurement. The PM_2.5_ concentration data were downloaded using Windows software (Grimm 1.177 ver. 3.0).

A total of 45 person-days of exposure data were collected in summer 2013. The field technicians simulated the time–activity patterns of selected residents of Seoul, who recorded their activity schedules in a diary. The mean ambient PM_10_ concentration in Seoul during the study period was 34.8 ± 18.0 µg/m^3^, which was below the national air quality standard of 100 µg/m^3^.

### 2.2. Time–Activity Patterns

Time–activity pattern data for 8072 subjects in Seoul were obtained from the Time Use Survey of Statistics Korea (KoSTAT), which was conducted in the summer of 2004. Details of the survey were presented in a previous study [27]. Briefly, the study population was selected from among 850 areas across Korea according to a standardized classification, to ensure representativeness of the general Korean population. From the total of 12,750 households, 12,651 residents (≥10 years of age) were selected for participation in the survey. The activity diaries were completed by 8072 subjects in Seoul. The number of subjects surveyed on weekdays, Saturday, and Sunday were 4849, 1608, and 1618 subjects, respectively. Locations were recorded according to 10-min intervals and classified as follows: Residential indoor, transportation, and “other”. The microenvironments except own residential indoors and transportation were grouped into “other” in the Time Use Survey. The survey captured demographic, socioeconomic, and familial data.

### 2.3. Kosem-PM

The KoSEM-PM was developed to estimate daily PM_2.5_ exposure levels of the general population in Korea. For the first step of the model development, we used data of Seoul. In this study, microenvironmental measurements obtained in Seoul at 1-min intervals over 59,945 min, and time-activity patterns of Seoul residents obtained at 10-min intervals (as recorded in the Time Use Survey; 8072 person-days) were used. Measured PM_2.5_ concentrations were grouped into three microenvironment categories (residential indoors, transportation, and “other”) to match the Time Use Survey. For 24 h, PM_2.5_ concentrations were arranged in 10-min increment in the three microenvironments since the microenvironmental concentration would vary over time of the day. Overall, 409 PM_2.5_ concentration data sets out of 432 sets (144 10-min data sets × 3 microenvironments) were available for analysis.

#### 2.3.1. Estimation of Personal Exposure Levels of the 8072 Residents of Seoul

Personal exposure levels of the 8072 residents of Seoul were estimated using the Equation (1).

(1) $$Personal\;exposure=\overset{144}{\underset{t=1}{\sum }}C_{t\left(m\right)}/144$$

where *C_t(m)_* is the “mean” PM_2.5_ concentration of the microenvironment *m* (residential indoors, transportation, and “others”) in time *t*. *t* was based on 10 min interval of 24 h. Microenvironment *m* was determined by time activity pattern of the individual on 10 min interval.

#### 2.3.2. Simulation of Population Exposure to PM_2.5_

Equation (1) was used again for simulation of population exposure, however, *C_t(m)_* here was the “distribution” of PM_2.5_ concentration of the microenvironment *m* in time *t* on 10 min interval. The distribution of the each PM_2.5_ data set for the three microenvironments were obtained every 10 min. Each PM_2.5_ concentration data set was subjected to distribution fitting for accurate estimation of concentration distributions, with the best-fitted data being selected. Personal exposure was estimated by applying the PM_2.5_ concentration distribution data to the matching microenvironments at 10-min intervals. Ten thousand concentration values were randomly extracted from the distributions every 10 min and the exposure was calculated using these values. The exposure level at each 10-min interval was simulated by Monte-Carlo simulation, in which we set the number of trials to 10,000.

### 2.4. Statistical Analysis

A statistical analysis of the data was performed using R software (R Development Core Team, Vienna, Austria). To evaluate the trends in PM_2.5_ exposure levels by day of the week within the 8072 person-days data set, the data were further categorized into three groups: Weekdays, Saturdays, and Sundays. The weekday PM_2.5_ level was the average from Monday to Friday. To estimate the time spent indoors, analysis of variance (ANOVA) and Scheffé’s post-hoc test were used to compare differences among weekdays, Saturdays, and Sundays. To determine factors associated with exposure, collinearity was tested using the variation inflation factor and a stepwise approach.

The high exposure group comprised all individuals within the top 5% regarding level of PM_2.5_ exposure; their characteristics were compared to those of the rest of the population. Association between this exposure groups and categorical (demographic) variables were analyzed using Fisher’s exact test of independence and t-test for categorical and continuous variables, respectively. Variables with a *p*-value of less than 0.1 were included as covariates in the final logistic regression model for exposure groups. A *p*-value < 0.05 was taken to indicate statistical significance.

## 3. Results

### 3.1. Microenvironmental PM_*2.5*_ Concentrations

**Figure 1:**
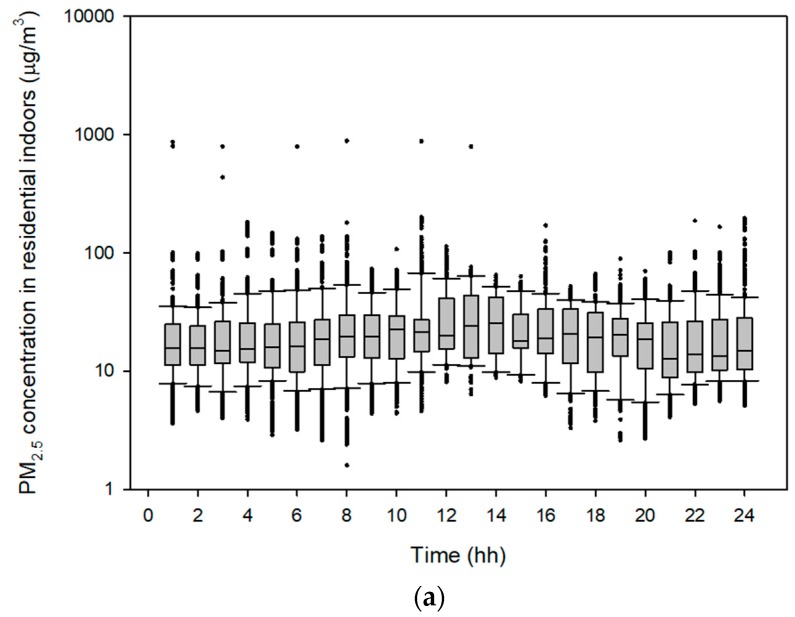

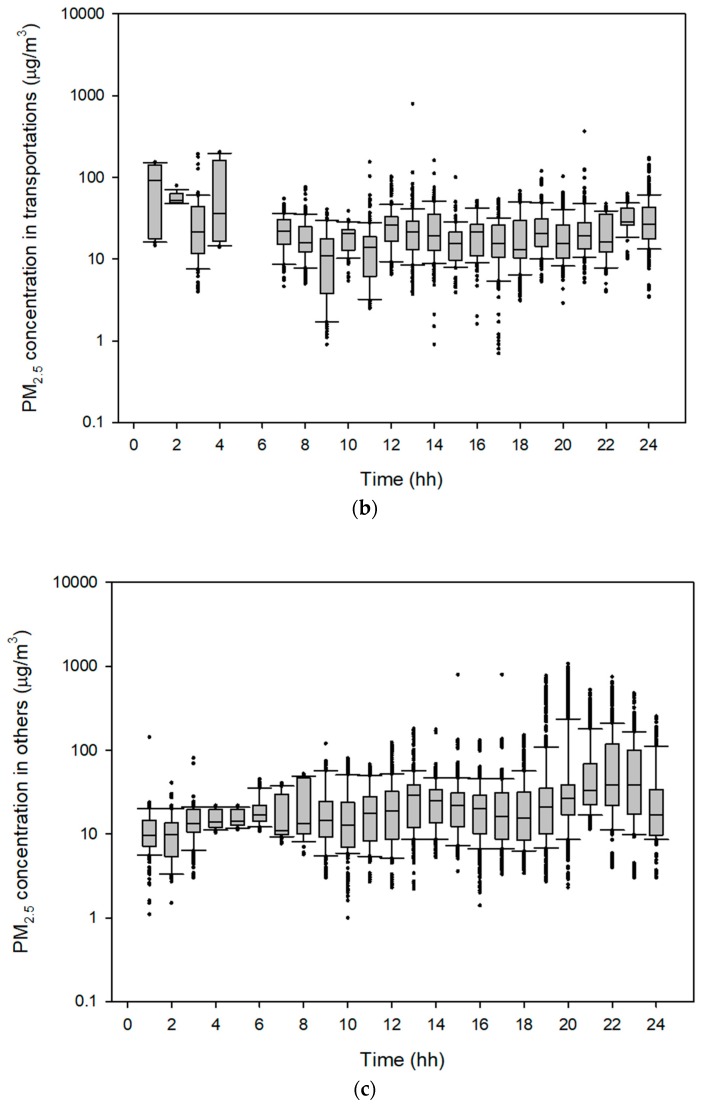
Fine particulate matter (PM_2.5_) concentrations in: (**a**) The residential indoors, (**b**) transportation, and (**c**) “other” microenvironment categories. Each bar represents the concentration in 1 h interval. The figures with every 10 min interval are provided in supplement (Figure S1).

Data for microenvironmental PM_2.5_ concentrations measured over 59,945 min (about 41.6 days) were included in the simulation. The mean microenvironmental PM_2.5_ concentrations were highest in the “other” microenvironments category, followed by the transportation and residential indoors categories. The mean PM_2.5_ concentration in the residential indoors category was 23.7 ± 24.1 µg/m^3^ (range: 1.6–888.8 µg/m^3^; n = 32,984). The mean PM_2.5_ concentration in the transportation category was 24.2 ± 22.8 µg/m^3^ (range: 0.7–96.9 µg/m^3^; *n* = 5300). The mean PM_2.5_ concentration in the “other” microenvironment category was 34.7 ± 62.3 µg/m^3^ (range: 1.0–1078.6 µg/m^3^; *n* = 21,660). PM_2.5_ concentrations of 1 h interval for the three microenvironment categories are shown in Figure 1. PM_2.5_ concentrations of 10 min interval for the three microenvironment categories are presented in Figure S1.

### 3.2. Time–Activity Patterns of the 8072 Residents of Seoul

**Figure 2:**
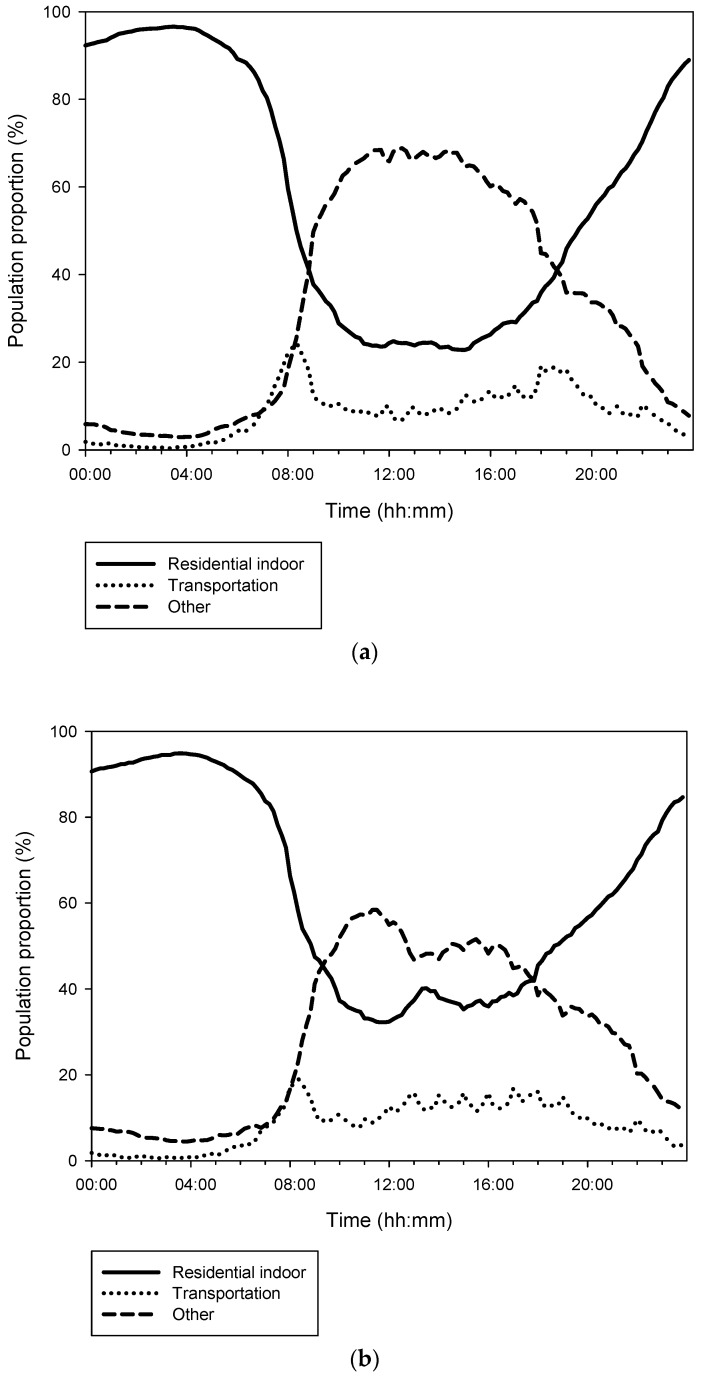

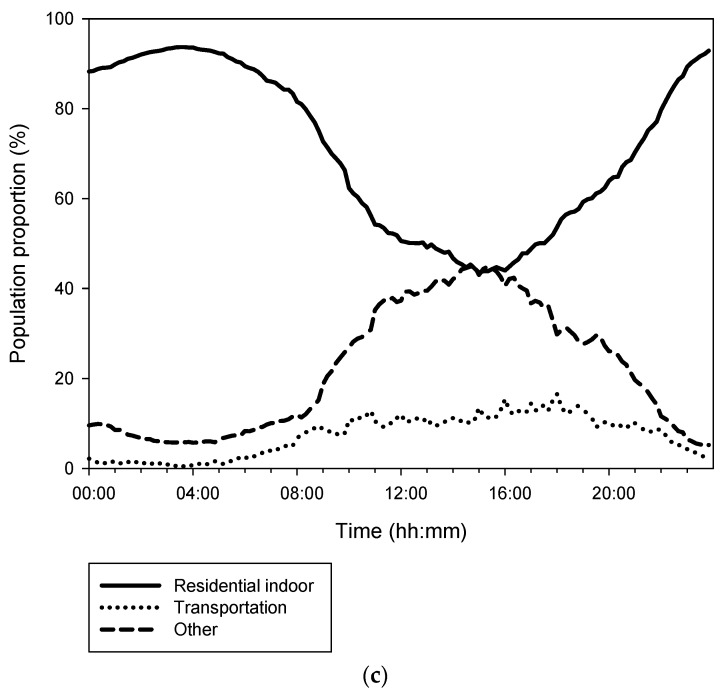
Time–activity patterns of the surveyed residents of Seoul on (**a**) weekdays, (**b**) Saturdays, and (**c**) Sundays.

Time–activity patterns of the surveyed residents differed by day of the week, as shown in Figure 2. On weekdays, the time spent in the residential indoors, transportation, and “other” microenvironments was 13.95 ± 4.77 h, 2.03 ± 1.68 h, and 8.01 ± 4.28 h, respectively. Approximately 20% of the surveyed residents stayed at home during the day time. On Saturdays, the respective values were 15.02 ± 5.09 h, 2.02 ± 1.72 h, and 6.96 ± 4.46 h, and approximately 30% of the surveyed residents remained at home during the day time. On Sundays, the respective values were 17.01 ± 5.40 h, 1.76 ± 1.86 h, and 5.23 ± 4.46 h, respectively, and more than 40% of the surveyed residents stayed at home all day. The time spent in the residential indoors, transportation, and “other” microenvironments differed significantly by day of the week (*p* < 0.001). Demographic characteristics, including gender, age, marital status, education, industry sector, job type, and monthly income were similar among the day of the week categories.

### 3.3. Personal Exposure Levels of the 8072 Residents of Seoul

**Figure 3:**
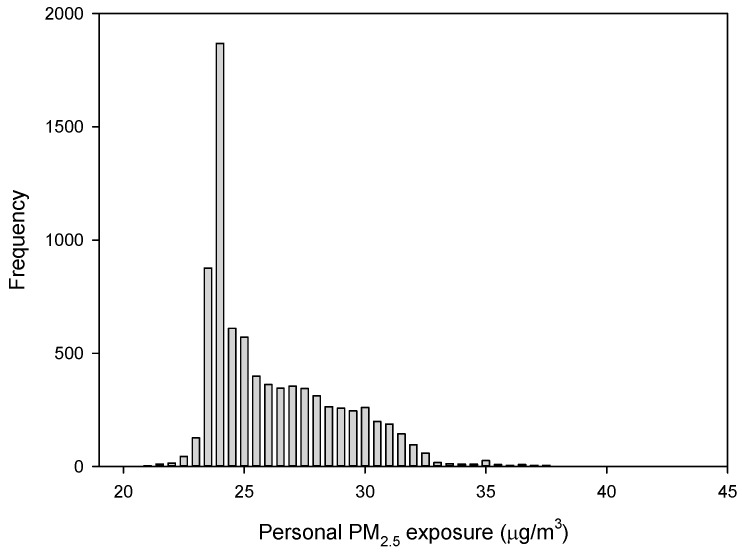
Daily personal PM_2.5_ exposure levels (µg/m^3^) of 8072 residents of Seoul.

Individual PM_2.5_ exposure levels were calculated according to the average PM_2.5_ concentration at each time interval. The mean personal PM_2.5_ exposure level of the surveyed residents was 26.0 ± 2.7 µg/m^3^ (range: 21.0–40.2 µg/m^3^; median = 25.0 µg/m^3^; 95th percentile = 31.1 µg/m^3^, 99th percentile = 33.8 µg/m^3^). The personal PM_2.5_ exposure levels of the 8072 residents of Seoul are shown in Figure 3. The data showed a log-normal distribution, as confirmed by a Q-Q plot. The geometric mean personal PM_2.5_ exposure level was 25.8 ± 1.1 µg/m^3^.

The personal PM_2.5_ exposure in high exposure group ranged from 31.1 µg/m^3^ to 40.2 µg/m^3^, with an average of 32.6 ± 1.7 µg/m^3^. The personal PM_2.5_ exposure in low exposure group ranged from 21.0 µg/m^3^ to 31.1 µg/m^3^, with an average of 25.6 ± 2.3 µg/m^3^. Univariate analysis revealed that the factors associated with high exposure were sex, education, industry sector, job type, working hours, house size, and house ownership status (all *p* < 0.05; Table 1). The high exposure group contained more males, more individuals with a higher education qualification, more tertiary sector and non-office workers, and more individuals with long working hours (all *p* < 0.001). The high exposure group had smaller houses and were less likely to own their own house (both *p* < 0.05).

The results of the multivariate logistic regression model for exposure groups are shown in Table 2. Five factors were significantly associated with high PM_2.5_ exposure levels: day of the week, age, industry sector, job type, and working hours. Among these factors, industry sector, job type, and working hours were the most significant. The individuals surveyed on Saturdays were more likely to be in the high exposure group than those surveyed on weekdays. Individuals surveyed on Sundays were less likely to be in the high exposure group, but not significant. Younger non-office-workers with longer working hours were more likely to be in the high exposure group, as were tertiary industry workers. Although not significant, sex, education level, monthly income, and house size were included in the model. Females, individuals with a higher monthly income, and those living in larger houses were less likely to be in the high exposure group. Compared to individuals with an educational level below middle school, those with a university level qualification or above were more likely to be in the high exposure group. Individuals with a monthly income below $2000, and those living in larger houses, were more likely to be in the high exposure group.

### 3.4. Simulated Population Exposure to PM_2.5_

**Figure 4:**
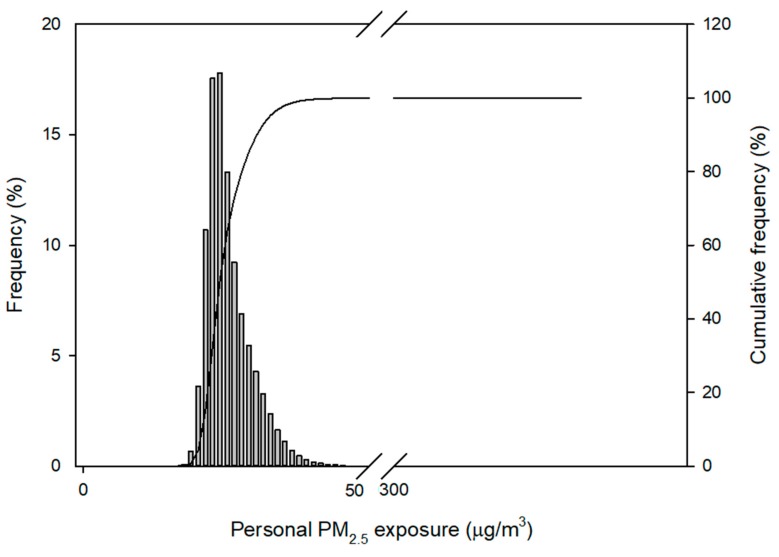
Personal exposure levels to PM_2.5_ (µg/m^3^).

Population exposure to PM_2.5_ was simulated according to the PM_2.5_ concentrations at each time interval. After 10,000 simulations, the mean population exposure to PM_2.5_ of the 8072 surveyed residents of Seoul was 25.5 ± 4.0 μg/m^3^ (range: 15.0–328.5 μg/m^3^; median = 24.5 µg/m^3^; 95th percentile = 33.2 µg/m^3^, 99th percentile = 37.9 µg/m^3^). The simulated personal PM_2.5_ exposure levels are shown in Figure 4.

## 4. Discussion

### 4.1. Microenvironmental PM_*2.5*_ Concentrations

The hourly PM_2.5_ concentrations differed among microenvironments. The microenvironments used in this model were classified into only three categories because the time-activity patterns of the national Time Use Survey had the three microenvironments. The Time Use Survey was conducted for economic activity. Although microenvironmental classification is limited, application of national data for environmental study is significant. There was little variation in hourly PM_2.5_ concentrations in the residential indoors microenvironment, wherein the PM_2.5_ concentration was high at lunch time (12:00–12:59). In the “other” microenvironment category, the PM_2.5_ concentrations were high in the evening and at night time (18:00–22:59). This was because this category included restaurants and bars. The microenvironmental PM_2.5_ concentration in the residential indoors category was similar to that in the transportation category. Some studies have reported a higher PM_2.5_ concentration in the transportation versus residential indoor microenvironment category [28,29,30]; in our study, this was probably because walking was included within the transportation category. The Time Use Survey since 2014 classified the microenvironments as follows: Own home, workplace/school, restaurant, other, walk, private transportation, and public transportation. Future studies could apply this approach with more diverse microenvironments.

### 4.2. Time-Activity Pattens of the Surveyed Residents

The residents of Seoul surveyed in this study spent 58.3% of their time in residential indoor environments, which was a similar proportion to that of the whole Korean population on weekdays (59.3%) [31]. The time spent in residential indoor environments by the citizens of Hong Kong (58.0%) was similar to that of Koreans [32]. Compared to western countries, Koreans spend less time in residential indoor environments [33,34,35,36,37,38]. A study conducted in the United States showed that Americans spent 70.9% of their time in residential indoor environments [39].

The time–activity patterns of our Seoul population varied by day of the week. Both morning and evening commuting patterns were observed on weekdays. On weekends, people spent more time in residential indoor environments than on weekdays. The time spent in “other” microenvironments was longest on weekdays, followed by Saturdays and Sundays; the opposite trend was seen for the residential indoor microenvironment category.

### 4.3. Personal PM_*2.5*_ Levels of Surveyed Residents

The individual exposure levels of the 8072 residents of Seoul were calculated based on their time–activity patterns and the PM_2.5_ concentration measured in each microenvironment at the time. The mean personal PM_2.5_ exposure level of the 8072 residents was 26.0 ± 2.7 µg/m^3^. However, this might have been an underestimation; microenvironmental concentrations were measured only when ambient PM_10_ concentrations were below the air quality standard of 100 µg/m^3^ (24-h average). Korean PM_2.5_ air quality standard was not available when the air quality was measured. In addition, measurements were taken with the aim of avoiding smokers where possible, in both indoor and outdoor environments. The main reason for calculating the personal exposure of the 8072 residents was to determine the factors associated with their exposure levels. The mean ambient PM_10_ concentration during the study period was 34.8 ± 18.0 µg/m^3^. Because the Korean government only began measuring PM_2.5_ in 2015, PM_2.5_ data could not be obtained for the study period.

For small proportion of the surveyed residents (511/8072; 6.3%), daily PM_2.5_ exposure levels were calculated by excluding the time spent in transit. It was because the microenvironmental PM_2.5_ concentrations in transit were not available for certain time, such as early morning (04:00–05:59) since we followed the time-location scenarios made from the Time Use Survey. These residents were in transit at least once at that time. If a person spent 20 min in transit between 04:00–05:59, their exposure level was calculated based on a period of 1420 min rather than 1440 min. The excluded data accounted for 0.13% of the total time period and thus could be neglected.

We grouped individuals with the highest PM_2.5_ exposure levels and determined the factors associated with their exposure. Day of the week, age, industry sector, job type, and working hours were all significantly associated with high PM_2.5_ exposure levels. In the EXPOLIS-Helsinki study, the most important sociodemographic factors associated with personal 48-h PM_2.5_ exposure levels were occupational status and educational level [40]. Occupational status, educational attainment, and age showed negative associations with exposure levels [41]. Environmental tobacco smoke (ETS) is the factor most strongly associated with PM exposure. We did not collect ETS data because the Time Use Survey was not designed to examine PM exposure levels, but rather how citizens use their time.

### 4.4. Simulated Population Exposure

This study developed a probabilistic simulation model to estimate the PM_2.5_ exposure levels of surveyed residents of Seoul. When the population exposure levels were simulated 10,000 times, the range of exposure level widened. It was found that 2.8% of the study population had personal exposure levels that exceeded the Korean PM_2.5_ air quality standards (24-h average) of 35 μg/m^3^, even though the outdoor PM levels were relatively low; this implies that high indoor PM_2.5_ concentrations contributed significantly to the daily exposures.

Some studies have estimated daily PM_2.5_ exposures using central-site monitoring data. Simulated annual average PM_2.5_ exposure levels ranged from 109 to 125 μg/m^3^ in New Delhi [42]. A case study conducted in Philadelphia, PA reported that the median daily PM_2.5_ exposure was 20 μg/m^3^ by using the Stochastic Human Exposure and Dose Simulation (SHEDS-PM) model [21]. In the EXPOLIS study, the cumulative average simulated 48-h population exposure levels in Athens, Prague, Basel, and Helsinki were 43, 37, 25, and 13 μg/m^3^, respectively [40]. Unlike the current study, the indoor microenvironmental concentrations used in these studies were calculated based on outdoor PM_2.5_ concentrations using mathematical modeling. The current study used directly measured microenvironmental concentrations to estimate population exposure levels.

### 4.5. Limitations

Application of actual microenvironmental measurement on personal exposure model was a key component of KoSEM-PM. Although 59,945 data points of microenvironmental measurements were applied in this model, they had limitation for generalization. Especially, this study included the microenvironmental measurement only when outdoor concentrations were complied with Korean ambient air quality standard of 100 μg/m^3^ for PM_10_. Since indoor concentration could be affected by ambient concentration, interpretation of the findings should be limited to relatively low ambient concentration condition. In addition, the data included only the weekday microenvironmental concentrations because only personal PM_2.5_ exposures collected during the weekday was available. This model could not be used to estimate actual population exposure in all situations. The model should be expanded with more data on high concentration days and weekends.

## 5. Conclusions

Daily personal PM_2.5_ exposure levels were determined using national time–activity data and directly measured PM_2.5_ concentrations in each microenvironment at the time. The PM_2.5_ exposure levels varied by exposure factors. A probabilistic simulation model was developed and estimated the exposure to PM_2.5_ of surveyed residents of Seoul, Korea. The KoSEM-PM might be a useful tool for estimating population exposure levels to other regions in Korea. To expand the use of KoSEM-PM, microenvironmental measurement data from other cities in Korea is required.

## Figures and Tables

**Table 1:** Characteristics of the high and low fine particulate matter (PM_2.5_) exposure groups.

Variables	Low Exposure Group *n* = 7668	High Exposure Group *n* = 404	*p*-Value
Day of the week—no. (%)			0.003 ^1^
Weekdays	4630 (60.4)	219 (54.2)	
Saturdays	1497 (19.5)	108 (26.7)	
Sundays	1541 (20.1)	77 (19.1)	
Sex—no. (%)			<0.001 ^1^
Male	3563 (46.5)	223 (57.7)	
Female	4105 (53.5)	171 (42.3)	
Age, years—median (range)	36 (10–93)	35 (11–87)	0.655
Marriage status—no. (%)			0.473
Married	4259 (55.5)	217 (53.7)	
Unmarried	3409 (44.5)	187 (46.3)	
Education—no. (%)			<0.001 ^1^
Middle school and below	2156 (28.1)	76 (18.8)	
College and below	2778 (36.2)	166 (41.1)	
University and above	2734 (35.7)	162 (40.1)	
Industry—no. (%)			<0.001 ^1^
Primary and secondary industry	741 (18.0)	25 (8.1)	
Tertiary industry	2227 (54.1)	205 (66.3)	
Other	1151(27.9)	79 (25.6)	
Job—no. (%)			<0.001 ^1^
Office worker	1933 (46.9)	91 (29.4)	
Non-office worker	2186 (53.1)	218 (70.6)	
Working hours, hour per week—median (range)	18 (0–120)	49 (0–105)	<0.001 ^1^
Monthly income—no. (%)			0.100
<$2000	6534 (85.2)	332 (82.2)	
≥$2000	1134 (14.8)	72 (17.8)	
House size, m^2^—median (range)	66.1 (9.9–337.2)	59.5 (16.5–198.3)	0.025 ^1^
Own house—no. (%)			0.002 ^1^
Yes	4025 (52.5)	179 (44.3)	
No	3643 (47.5)	225 (55.7)	
Own car—no. (%)			0.595
Yes	4900 (63.9)	46 (11.4)	
No	2768 (36.1)	358 (88.6)	

^1^*p* < 0.05.

**Table 2:** Results of the multivariate logistic regression model of the high PM_2.5_ exposure group.

Variables	Coefficient	Standard Error	*p*-Value ^1^	OR ^3^ (95% CI)
Day of the week			0.044 ^2^	
(Weekdays)				
Saturdays	0.356	0.147		1.428 (1.701–1.903)
Sundays	−0.017	0.162		0.983 (0.715–1.350)
Sex			0.240	
(Male)				
Female	−0.156	0.133		0.856 (0.660–1.110)
Age	−0.019	0.006	0.002 ^2^	0.981 (0.969–0.993)
Education			0.173	
(Middle school and below)				
College and below	−0.148	0.190		0.863 (0.595–1.251)
University and above	0.139	0.232		1.149 (0.729–1.810)
Industry			<0.001 ^2^	
(Primary and secondary industry)				
Tertiary industry	1.063	0.220		2.894 (1.880–4.454)
Other	0.843	0.239		2.324 (1.454–3.716)
Job			<0.001 ^2^	
(Office worker)				
Non-office worker	0.797	0.161		2.220 (1.618–3.044)
Working hours	0.028	0.003	<0.001 ^2^	1.029 (1.022–1.036)
Monthly income			0.199	
(<$2000)				
≥$2000	−0.202	0.159		0.817 (0.599–1.115)
House size	0.001	0.003	0.765	1.001 (0.996–1.006)
Own house			0.425	
(Yes)				
No	0.108	0.136		1.114 (0.854–1.454)

^1^*p*-Value by likelihood ratio test (LRT); ^2^*p* < 0.05; ^3^ OR = odds ratio = Odds_(high)_/Odds_(low)_; CI = confidence interval; items in brackets: reference.

## Supplementary Materials

The following is available online at http://www.mdpi.com/1660-4601/15/12/2808/s1, Figure S1: Fine particulate matter (PM_2.5_) concentrations in: (**a**) the residential indoors, (**b**) transportation, and (**c**) “other” microenvironment categories. Each bar represents the concentration in 10 min interval.
