# Supplementary material for: Characterization of a High PM2.5 Exposure Group in Seoul Using the Korea Simulation Exposure Model for PM2.5 (KoSEM-PM) Based on Time–Activity Patterns and Microenvironmental Measurements
Source: Int J Environ Res Public Health. 2018 Dec 10;15(12):2808. doi: 10.3390/ijerph15122808 (PMC6313682; doi:10.3390/ijerph15122808)
Supplement: Supplementary file 1 [file ijerph-15-02808-s001.pdf]

Supplementary Figure

Characterization of a High $PM_{2.5}$ Exposure Group in Seoul Using the Korea Simulation Exposure Model for $PM_{2.5}$ (KoSEM-PM) based on Time–Activity Patterns and Microenvironmental Measurements

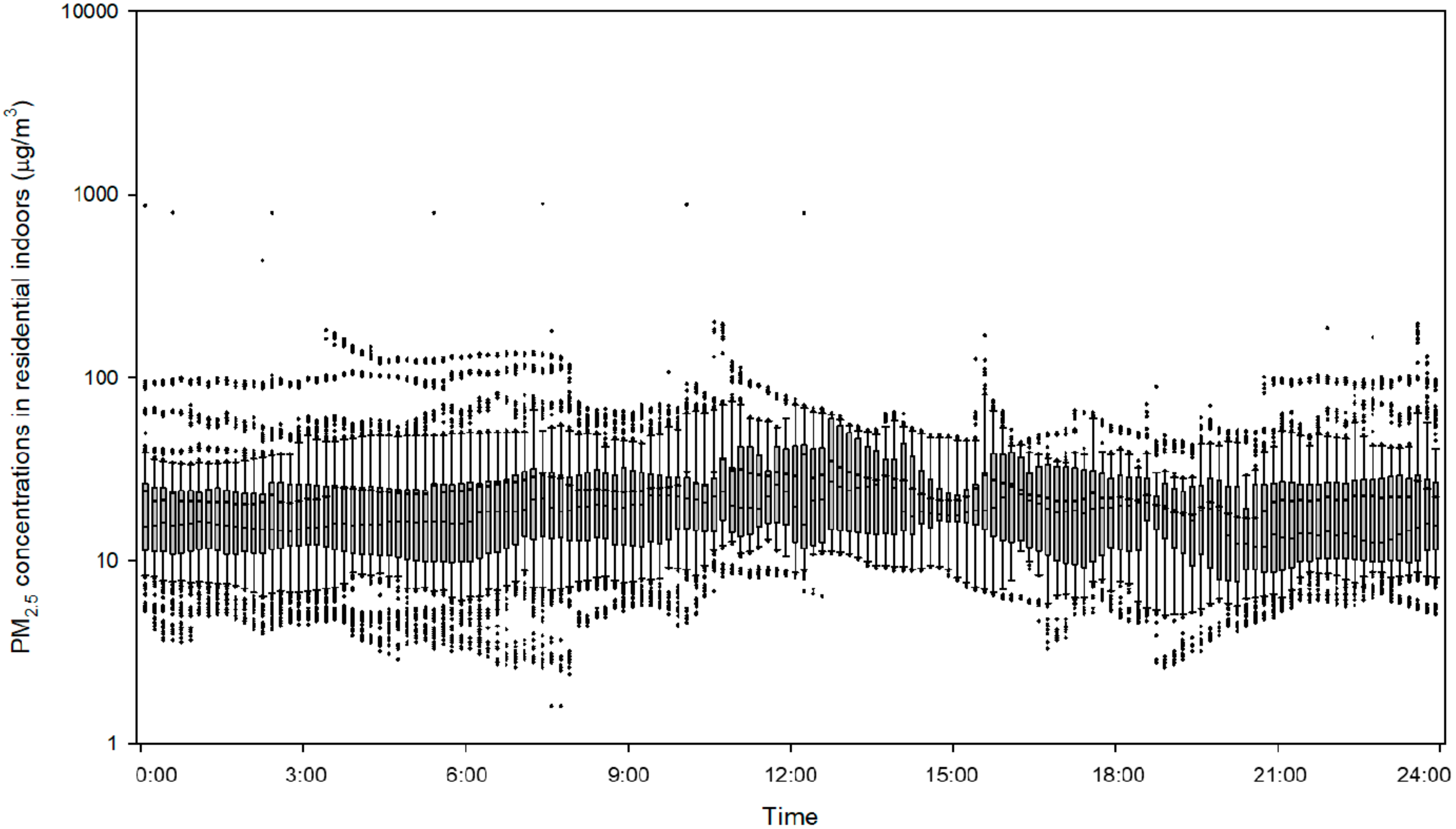

PM2.5 concentrations in residential indoors (μg/m3)
10000
1000
100
10
1
0:00
3:00
6:00
9:00
12:00
15:00
18:00
21:00
24:00
Time

(**a**)

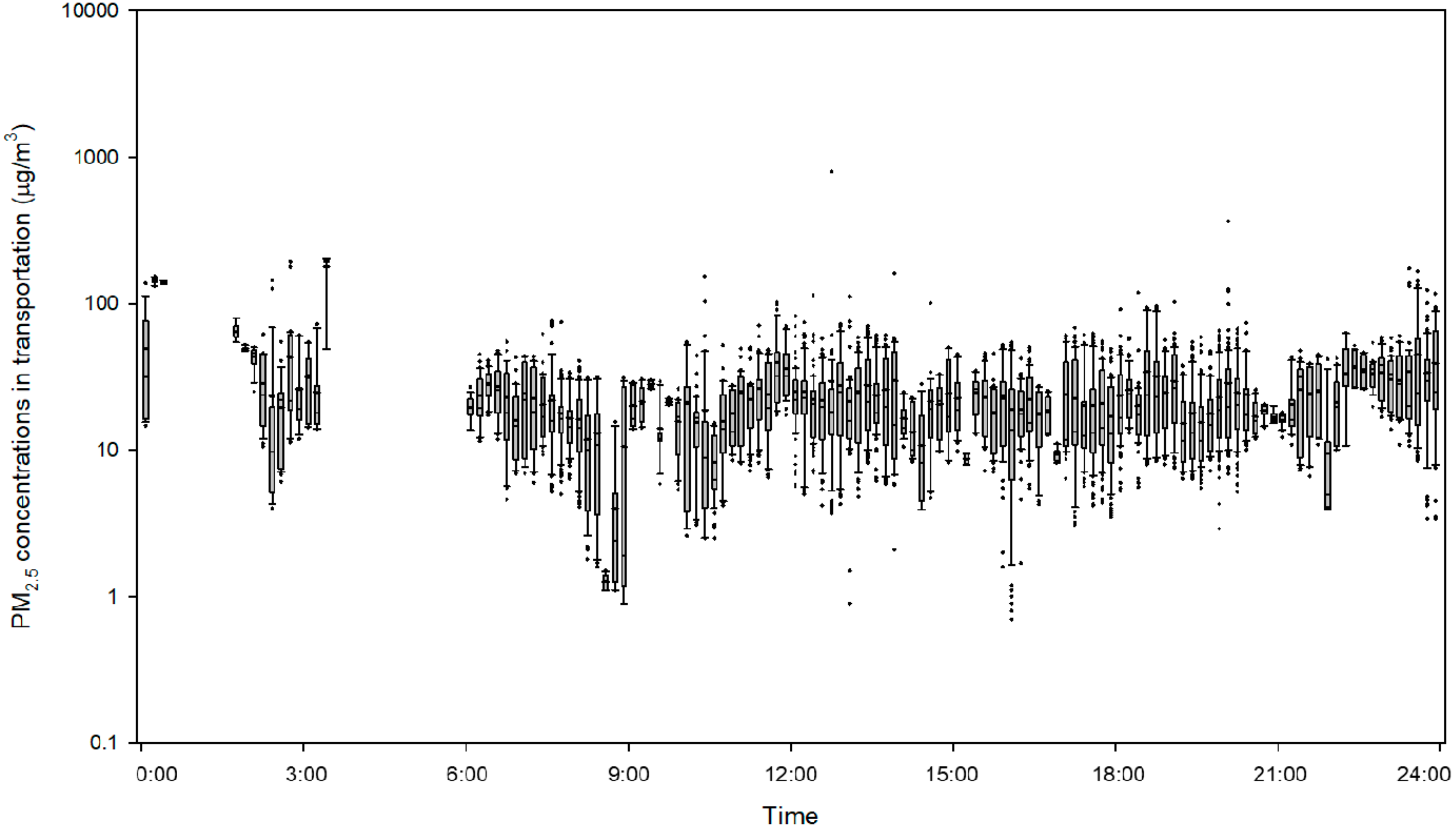

10000
1000
100
10
1
0.1
PM2.5 concentrations in transportation (μg/m3)
0:00
3:00
6:00
9:00
12:00
15:00
18:00
21:00
24:00
Time

(**b**)

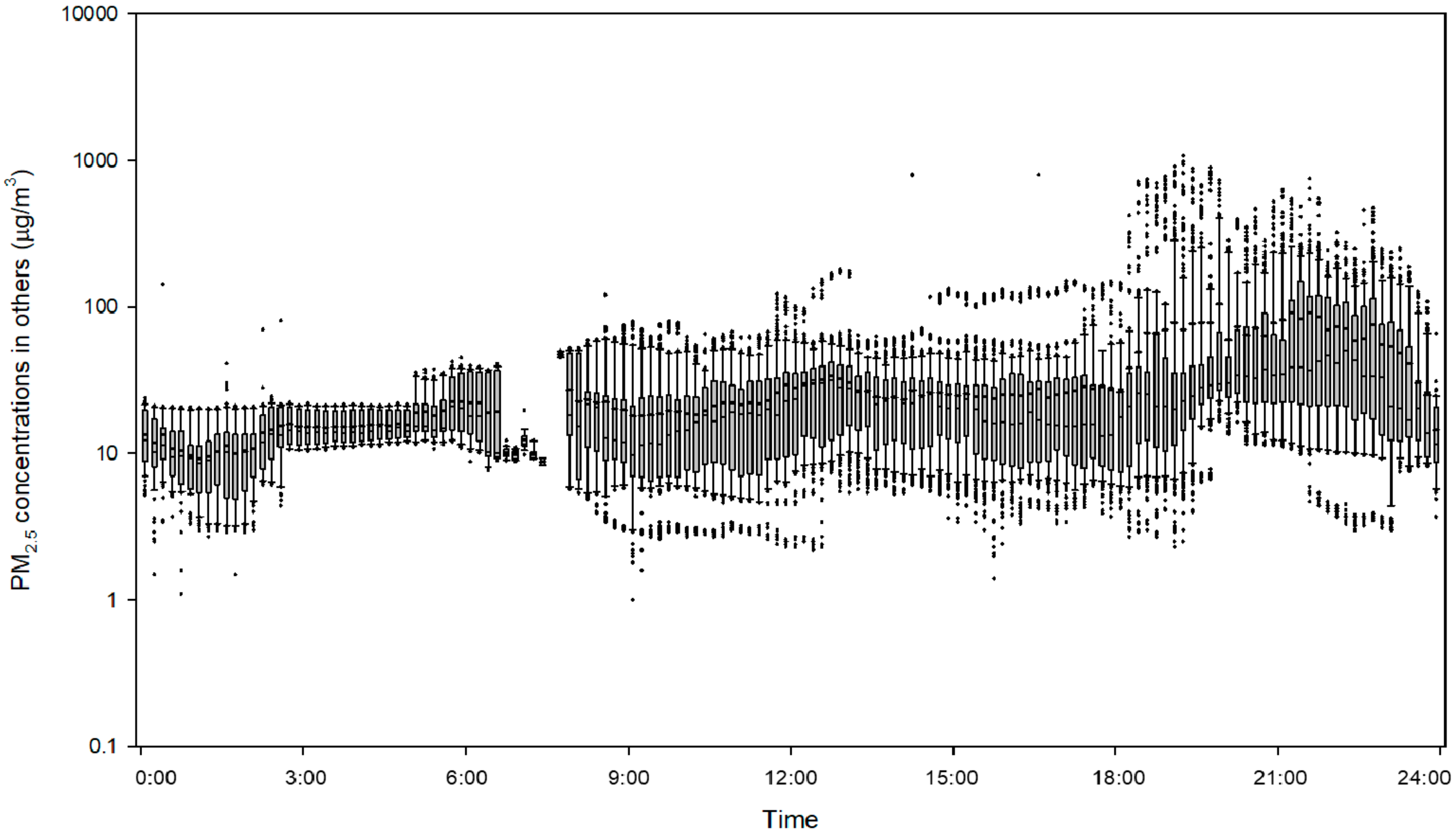

(c)

Figure S1. Fine particulate matter ($PM_{2.5}$) concentrations in: (**a**) the residential indoors, (**b**) transportation, and (**c**) "other" microenvironment categories. Each bar represents the concentration in 10 min interval.
